# Photovoltaic Device Based on Monolayer Compositionally Graded Transition Metal Dichalcogenide Alloy

**DOI:** 10.1002/smtd.202501997

**Published:** 2026-02-12

**Authors:** Hao Ou, Sota Tsukamoto, Tenta Kitamura, Motoki Matsuno, Koshi Oi, Togo Takahashi, Takahiko Endo, Yasumitsu Miyata, Jiang Pu, Taishi Takenobu

**Affiliations:** ^1^ Department of Physics Institute of Science Tokyo Meguro Tokyo Japan; ^2^ Department of Applied Physics Nagoya University Nagoya Japan; ^3^ Research Center for Materials Nanoarchitectonics National Institute for Materials Science Tsukuba Japan

**Keywords:** monolayer alloy, photoresponse, photovoltaic device, p‐n junction, transition metal dichalcogenide

## Abstract

Monolayer transition metal dichalcogenides (TMDCs) have been widely studied for the fabrication of photovoltaic devices with high energy conversion efficiencies for future ultrathin optoelectronic devices. To create efficient photovoltaic devices, in‐plane heterostructures, whose composition can be artificially tailored by chemical vapor deposition, are a promising approach to form p‐n junctions spontaneously. Although sharp in‐plane heterostructures are typically employed, their narrow heterointerfaces are prone to defect sensitivity and thermal losses, which can significantly reduce device performance. In this study, we demonstrated that the spontaneous p‐n junction devices fabricated based on chemically synthesized compositionally graded monolayer WS_2_ *_x_* Se_2(1‐_ *_x_*_)_ alloys exhibited enhanced photoresponse performance. By conducting photocurrent and photoluminescence mappings, we revealed the correlation between the photocurrent generation behavior and local composition gradient. Moreover, the monolayer alloy device exhibited an open‐circuit voltage as high as 0.66 V, highlighting the potential of a compositionally graded p‐n junction for high‐efficiency photovoltaic devices. Our study presents a new approach for the development of efficient TMDC‐based optoelectronic devices.

## Graphical abstract

Photovoltaic devices based on compositionally graded monolayer WS_2_ *_x_* Se_2(1‐_ *_x_*_)_ alloys were fabricated and characterized. A spontaneous p‐n junction was formed inside the alloy region, and significant photocurrent was collected. The devices exhibited high gate tunability with a large open‐circuit voltage. The simple structure and notable performance of these photovoltaic devices indicate the potential of our new approach for exploiting efficient optoelectronics based on synthesized monolayer alloys.

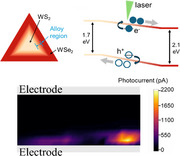

## 1 Introduction

Photovoltaic devices, such as solar cells that convert photons into electrical signals, are essential for the future energy conversion industry. As the demand for advanced solar cell technologies increases over the years, it becomes increasingly important to develop more efficient and functionalized photovoltaic devices. In recent years, atomically thin transition metal dichalcogenides (TMDCs) have attracted significant attention due to their low‐dimensional nature and strong light–matter interactions [1, 2]. Typical semiconducting TMDCs exhibit band gaps ranging from 1 to 2 eV, corresponding to photon energies ranging from nearly red to infrared, making them promising candidates for high‐performance solar cell components [3, 4, 5, 6]. For instance, monolayer TMDC‐based photovoltaic devices have been widely explored, showing pronounced photovoltaic effects, with an open‐circuit voltage (*V*_oc_) exceeding 0.8 V and photoconversion efficiency (PCE) greater than 50%, highlighting their potential for use in transparent and flexible solar cells [4, 7, 8, 9, 10]. However, existing methods used to fabricate p‐n junction devices often require relatively complex structures, such as split gate structures [5]. In addition, chemical methods require surface treatment or functionalization, which can compromise device performance and reproducibility [11, 12]. Although van der Waals stacking can spontaneously form p‐n junctions via appropriate selection of materials, it inevitably lacks practical scalability and reliability because of the manual fabrication process [13, 14, 15, 16].

To address these limitations, the direct growth of monolayer lateral heterostructures is regarded as a promising strategy for designing efficient photovoltaic devices. Chemically synthesized lateral heterostructures possess naturally formed p‐n junctions, enabling device fabrication by simply depositing electrodes in suitable locations [17, 18, 19, 20]. For example, Duan et al. demonstrated the epitaxial growth of a WS_2_‐WSe_2_ lateral heterostructure with a sharp interface separating the two material regions [17]. An obvious photocurrent response was observed near the interface, confirming efficient electron–hole separation. However, because the width of the interface is typically ∼1 nm [18], the device performance is highly sensitive to defects within this narrow region. These defects can disrupt the band alignment and local electric field, which degrades carrier generation and separation efficiency. Furthermore, the heterostructure only provides two discrete bandgap values, and therefore, the absorption of photons with a continuous energy distribution inevitably leads to thermalization losses, which reduce the efficiency of the device [21, 22].

To overcome these limitations, we focused on the recently proposed compositionally graded monolayer TMDC alloys [23, 24, 25]. Unlike the formation of a sharp atomic interface in typical lateral heterostructures, the atomic registry in graded alloys shows a gradual variation over several micrometers (µm). Consequently, the composition, band structure, and optical properties continuously vary inside the alloy regions. Indeed, we had demonstrated color‐tunable light‐emitting devices based on monolayer WS_2_ *_x_* Se_2(1‐_ *_x_*_)_ alloys [25]. However, there is a lack of detailed investigation of the photocurrent generation mechanism and demonstration of the photovoltaic device using such compositionally graded alloys. Hence, in this study, we examined the photoresponse of a two‐terminal monolayer WS_2_ *_x_* Se_2(1‐_ *_x_*_)_ device. Correlated photoluminescence and photocurrent mapping revealed the photovoltaic effect inside the alloy region, in which the p‐n junction formed spontaneously, resulting from the spatially varying atomic composition. We demonstrated the photovoltaic effect in a simple device structure with a high *V*_oc_ (∼0.66 V), which exceeded the typical values of monolayer or heterostructure‐based photovoltaic devices. Their simple structure and notable performance indicate the great potential of compositionally graded TMDC alloys for the fabrication of efficient photovoltaic devices.

## 2 Results and Discussion

### 2.1 Spontaneous Formation of p‐n Junction in Monolayer WS_2x_Se_2(1‐x)_

**FIGURE 1:**
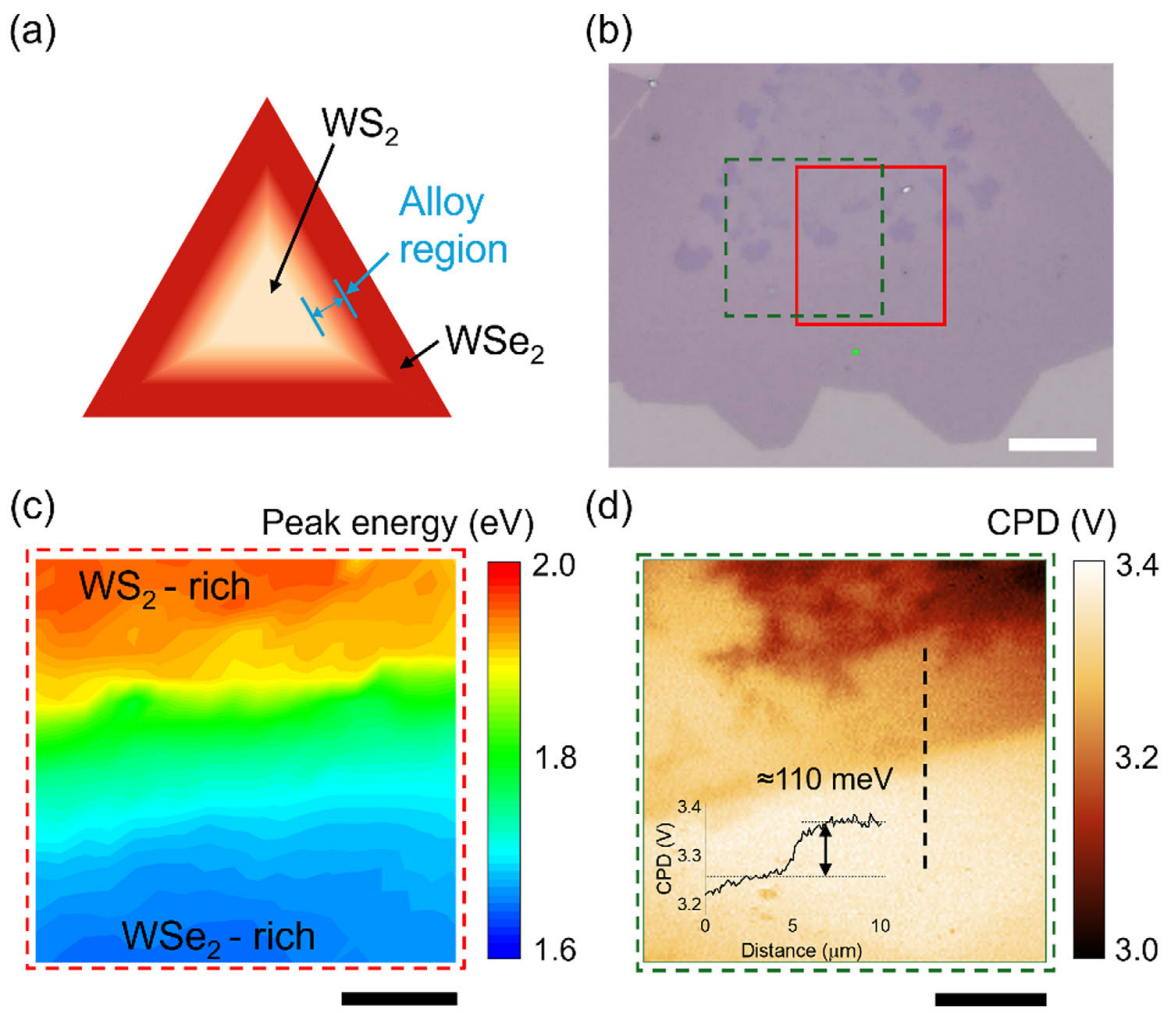
Characterization of monolayer compositionally graded WS_2_ *_x_* Se_2(1‐_ *_x_*_)_ alloy. (a) Schematic of the composition variation in the monolayer alloy. (b) Optical image of a CVD‐grown monolayer alloy. The scale bar is 10 µm. The green dot denotes the laser spot. The red and green rectangles indicate the regions on which (c) PL peak energy mapping and (d) surface potential mapping were carried out, respectively. The inset of (d) shows the CPD profile along the dashed line. The scale bars are 5 µm.

The compositionally graded monolayer WS_2_ *_x_* Se_2(1‐_ *_x_*_)_ was synthesized by chemical vapor deposition (CVD), as reported in our previous study [25]. Although the synthesis process was essentially the same as that in our previous study, in this study, we primarily exploited the spontaneous band structure of the compositionally graded alloy and demonstrated photovoltaic carrier generation and separation under illumination. In addition, we performed atomic force microscopy (AFM) and spatial optical measurements to confirm the monolayer nature and composition‐dependent optical property variation of the synthesized sample (Figure S1). Based on optical measurements and first‐principle calculations, when the composition gradually changed from WS_2_ to WSe_2_, the band structure changed accordingly [25]. Given that WS_2_ is typically *n*‐type whereas WSe_2_ is *p*‐type [26, 27], a gradual p‐n junction is expected to form spontaneously within the alloy region (Figure 1a). To verify this, we measured the photoluminescence (PL) spectra and surface potential variation across the WS_2_ *_x_* Se_2(1‐_ *_x_*_)_ alloy region, as shown in Figure 1b. The surface potential variation was characterized by the contact potential difference (CPD) using Kelvin probe force microscopy (KPFM). The alloy region was identified by the PL peak energy map (Figure 1c), in which the peak energy gradually shifted from ∼2.0 to ∼1.65 eV from the upper part to the lower part, indicating a composition gradient from the WS_2_‐rich region to the WSe_2_‐rich region [28]. The CPD map is presented in Figure 1d, which reveals a clear variation in the surface potential. Notably, the surface potential contrast approximately followed the PL peak energy shift, suggesting a composition‐correlated surface potential landscape in the measured region. We obtained a surface potential difference of ∼110 meV between the WSe_2_‐rich and WS_2_‐rich regions (inset of Figure 1d). It shall be noted that even though multilayer islands were present in the sample (see Figure 1b), they did not spatially correlate with the PL (Figure 1c) and CPD (Figure 1d) variations because both figures exhibited gradual changes along the same direction, which was distinct from the multilayer islands. In particular, in the CPD map, the multilayer islands typically exhibited lower values, and thus, shadows could be observed (Figure 1d). The CPD signals in these islands appeared as abrupt changes, and one could identify them from the background of the gradient. Therefore, the observed variation originated from the composition gradient. In addition, we did not observe any phase separation in our samples [25], as confirmed by the spatial optical measurements shown in Figure S1. Furthermore, prior experimental studies and thermodynamic simulations have indicated the feasibility of CVD conditions for growing a miscible monolayer WS_2_ *_x_* Se_2(1‐_ *_x_*_)_ solid–solution alloy [29, 30].

**FIGURE 2:**
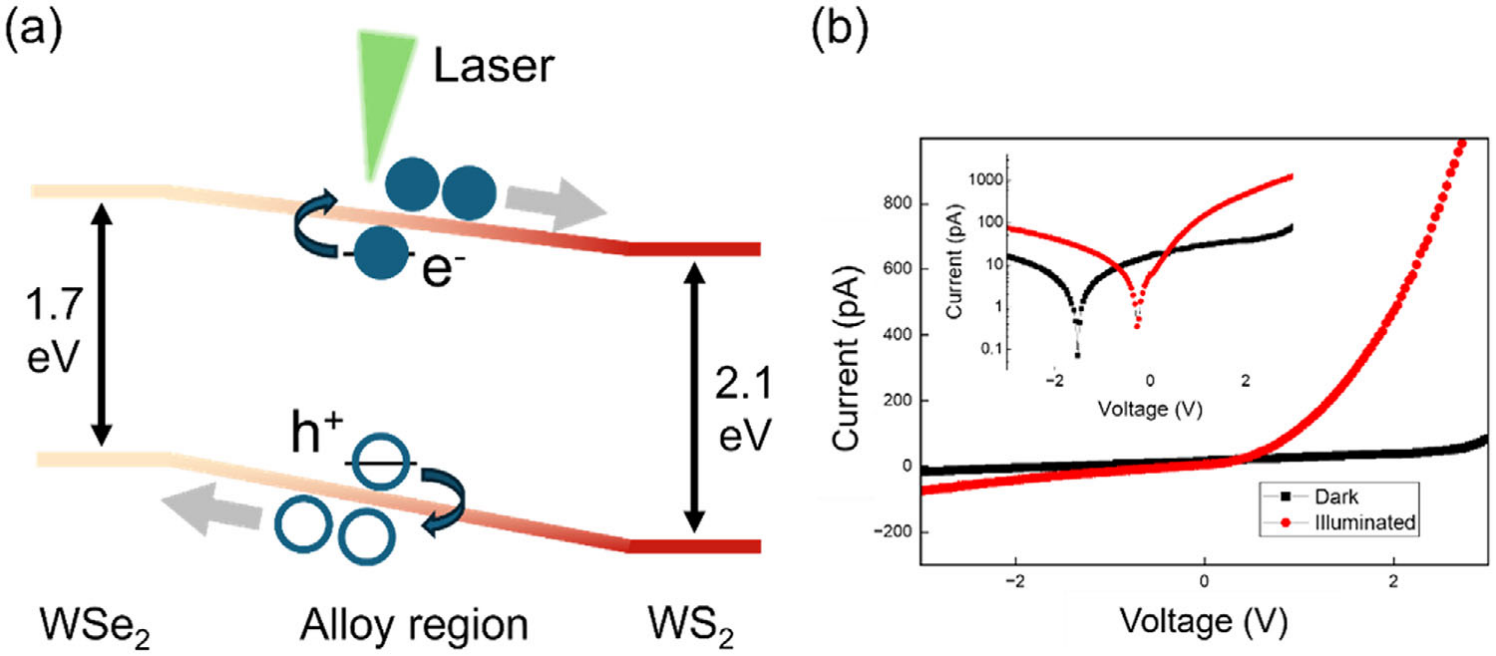
Gradual p‐n junction formation. (a) Schematic of the band diagram and photocurrent generation mechanism of the monolayer alloy. (b) Current–voltage (*I*–*V*) curves in the linear scale of the two‐terminal device (labeled as device #1) under dark and illuminated conditions. The inset of (b) shows the curves in the logarithmic scale.

Based on the results obtained from PL mapping, KPFM mapping, and previous theoretical calculations, we deduced a continually varying band structure across the alloy region, as schematically illustrated in Figure 2a. More importantly, the gradient of the surface potential indicated the presence of a built‐in electric field. When the alloy was illuminated, photogenerated electron–hole pairs were separated by this built‐in field, forming free carriers swept toward the WS_2_‐rich region (electrons) or WSe_2_‐rich region (holes), resulting in a photocurrent. To further investigate this behavior, we measured the two‐terminal current–voltage (*I*–*V*) relationship of the sample (labeled as device #1), where the channel region was aligned approximately parallel to the composition gradient. As shown in Figure 2b, the devices exhibited rectifying behavior in the absence of illumination (black line), strongly supporting the existence of a p‐n junction inside the sample. However, the *I*–*V* curve differed from that of a typical p‐n junction, as there appeared to be a linear increase in the current when the applied voltage was between 0 and +2 V. This may be due to the presence of intrinsic (undoped) regions of WS_2_ and WSe_2_ inside the channel, which introduce additional resistive components into the current. We then measured the *I*–*V* curve by illuminating the entire device with a 532‐nm laser (red line in Figure 2b). We observed an increase in the reverse current compared with the dark current. Simultaneously, the current increased rapidly under a forward bias, which was possibly due to the doping of intrinsic regions and reduced contact resistance under illumination. The illuminated curve can be explained by the modified Schockley diode equation in which the response of the current to the applied bias is still exponential, whereas the reverse saturation current is modified (see Section S2) [29]. The change in the *I*—*V* characteristics under illumination strongly indicated that the p‐n junction inside the alloy region exhibited an observable optoelectronic response, with a clear zero‐bias photocurrent (inset of Figure 2b), which deserved a detailed investigation of the photocurrent generation mechanism.

### 2.2 Spatial Photocurrent Mapping

**FIGURE 3:**
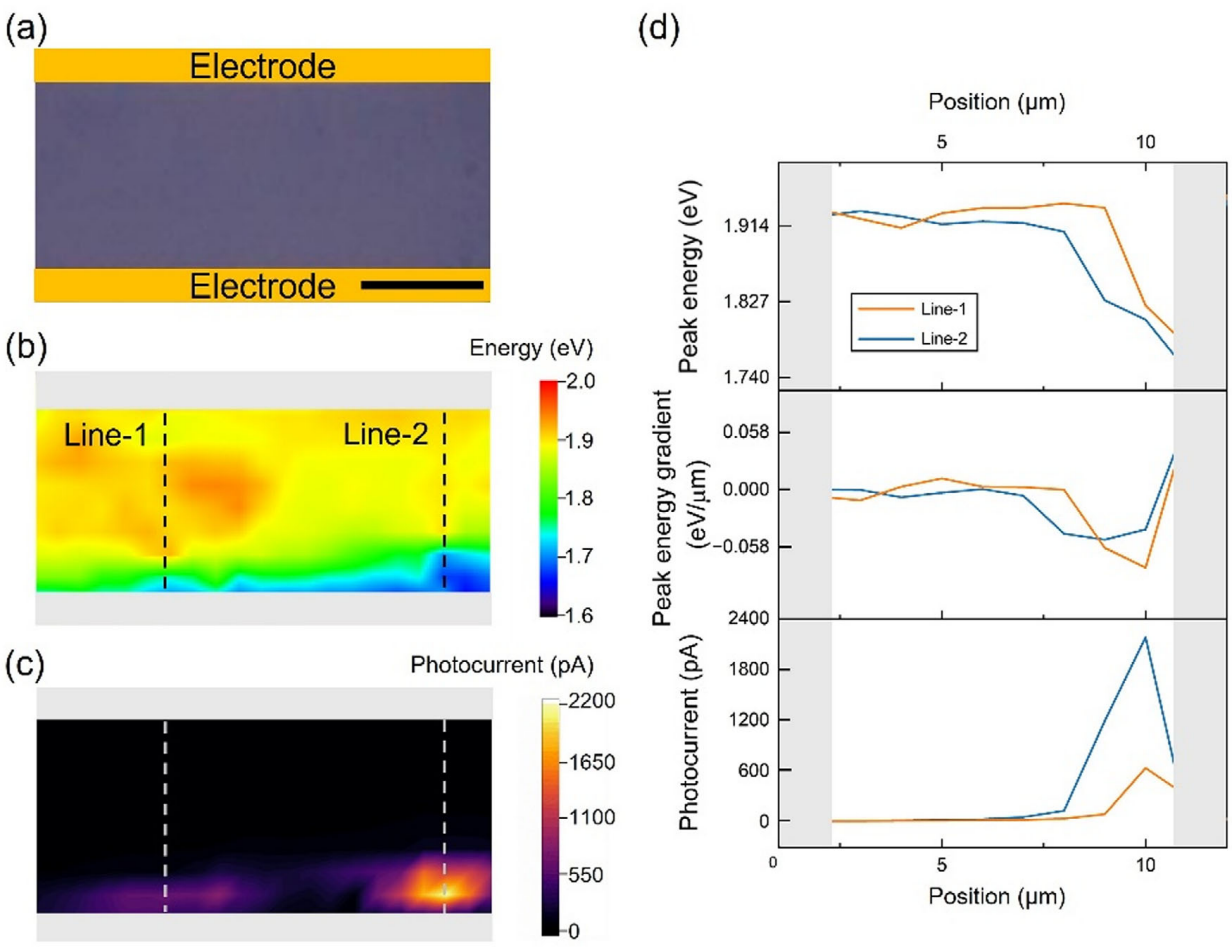
Photocurrent generation characterization of the monolayer alloy device. (a) Microscopic image of the channel region of device #2. The scale bar is 5 µm. (b) Corresponding PL map. (c) Corresponding photocurrent map. (d) Peak energy, peak energy gradient, and photocurrent profiles along the dashed lines in (b) and (c).

We then investigated the location‐dependent photocurrent generation of device #2. We defined the channel in the alloy region, which was free from any multilayer islands, to exclude their possible influence (Figure 3a). It shall be noted that we used a 100 × lens to limit the diameter of the illuminating laser spot to ∼2 µm for spatial photocurrent mapping, which was significantly smaller than that of a recently reported photoresponse study on alloy samples [23]. PL mapping was performed simultaneously with short‐circuit current mapping (*I_sc_*, where we did not apply bias during the measurement), as described in Methods. Therefore, we were able to correlate the local optical properties with the photocurrent generation behavior. Based on the results of PL mapping (Figure 3a), we observed a peak energy shift within the channel, indicating the existence of a composition gradient. More importantly, the corresponding photocurrent map revealed that when the alloy region was illuminated by the laser, a photocurrent as high as 2.2 nA was observed (Figure 3b). In contrast, we did not observe significant photocurrent in regions where the PL peak energy was nearly constant. Considering the existence of the p‐n junction inside this region, the collected photocurrent provides solid evidence of the photovoltaic response from the alloy region. It is worth noting that although the location of the generated photocurrent was close to the electrode, the Schottky junction between the electrode and sample could not be regarded as the origin because (1) the photocurrent could still be generated when the center of the focused laser spot was away from the electrode and the maximum photocurrent value was clearly not at the edge of the electrodes (Figure 3b), and (2) no photocurrent was observed when the laser spot was within proximity of the other electrode. These properties differed from those of typical Schottky junction‐based devices. We also excluded the possibility of the device being a phototransistor because it showed rectified transport behavior (Figure 2b), and no bias or gate voltage was applied during this measurement.

The photocurrent map of device #1 is shown in Figure S3, which had a longer channel length, and the alloy region was located near its center. The photocurrent map revealed a peak within the channel, located away from the electrodes. Thus, we confirmed again that a photocurrent was generated from the alloy region through the photovoltaic effect. The collected current value was only on the order of picoamperes (pA), which will be discussed later. The spontaneously formed, gradual p‐n junction enabled the sample to be fabricated into a photovoltaic device with a highly simple structure. Moreover, as a previous study unveiled [23], owing to the spatially varying band structure, the device showed photoresponse within a spectral range from nearly red to infrared illumination, highlighting its potential for high‐performance broadband optoelectronic applications.

### 2.3 Photocurrent Generation in the Gradual p‐n Junction

To examine the photocurrent generation behavior in detail, in Figure 3c, we plotted the spatial profiles of the PL peak energy, the gradient of the PL peak energy shift, and the corresponding photocurrent along the black dashed line in the PL map (Figure 3a) and the white dashed line in the photocurrent map (Figure 3b). The same analysis for device #1 is summarized in Figure S3. Here, we used the gradient of the PL peak energy shift to approximate the local built‐in field, as previous band structure calculations revealed that both the conduction band edge and valence band edge varied almost linearly with the composition variation [25]. Based on the profiles shown in Figure 3c, both the peak energy gradient and photocurrent exhibited peak behaviors, indicating the nonlinearity of the composition variation and the resulting spatial dependence of photocurrent generation. However, we found that the maxima of these two profiles did not necessarily coincide. This was particularly evident when the alloy region was relatively far from the electrodes (Figure S3). In an ideal p‐n junction, the built‐in field causes a drift of the photo‐excited excess carriers, leading to a drift current. Therefore, for a given laser power, it is reasonable to relate the maximum PL energy shift gradient to the maximum photocurrent value because the former indicates the local built‐in field magnitude and the latter is related to the short‐circuit current. However, this correspondence was not evident in our devices. The peak mismatch was more prominent in the device with a longer channel, in which the alloy region was located far away from the electrodes (Figure S3).

To address the above observations, we focused on the phenomenon in which photogenerated excess carriers moved under both drift and diffusion before reaching the electrodes. The carriers might recombine with their counterparts during migration, and hence, the recombined carriers did not contribute to the photocurrent. To illustrate this, we adopted the continuity equation of carrier density *n*, considering the drift term due to the built‐in potential, which is expressed as [30, 31]

(1) $$\frac{dn}{dt}=G+\frac{1}{q}\frac{d(J_{drift}+J_{diffusion})}{dx}-\frac{n}{\tau }$$

Here, *G* is the generation rate of the excess carriers. The current generation, due to carrier movement, consists of the drift current (*J_drift_*) and diffusion current (*J_diffusion_*) densities. The last term on the right‐hand side of Equation (1) indicates the recombination process of the carrier, where *τ* denotes the carrier lifetime. We numerically calculated the electron current distribution. For convenience, we considered a 1D condition and assumed that the electric field satisfied a Gaussian distribution according to the shape of the peak shift gradient (Figure 3c). The current density was calculated as a function of both laser spot positions, which shifted from the left side of the center of the built‐in field to the right side, and the channel length (represented by the location of the electrode). The details of the calculation are provided in Section S4.

**FIGURE 4:**
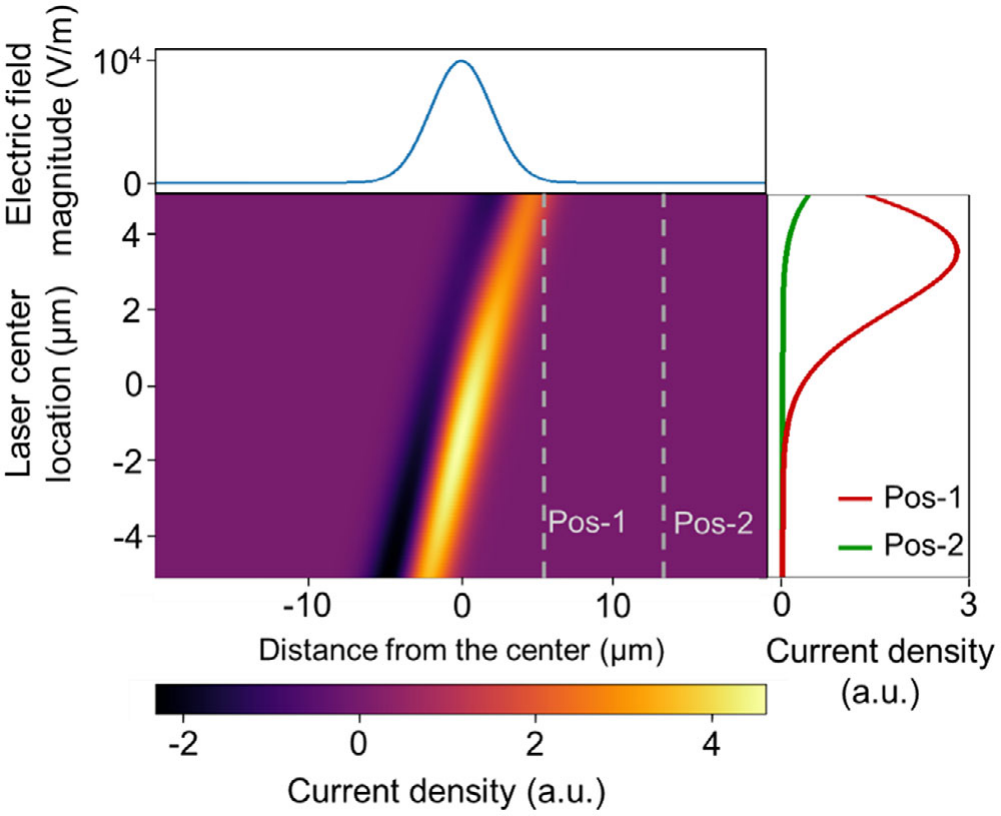
Calculated distance and laser position dependence of the photocurrent. The top panel indicates the built‐in electric field profile, and the right panel shows the photocurrent profile along the corresponding gray dashed lines in the main panel. The two dashed lines represent different locations of electrodes (see main text). It shall be highlighted that the laser center moves parallel to the channel and the built‐in electric field. The negative current density indicates that the direction of current is reversed.

The resulting photocurrent density map is shown in the main panel of Figure 4. The horizontal axis corresponds to the channel direction, and the vertical axis corresponds to the laser center position, which also shifts along the channel direction. The top panel shows the assumed Gaussian‐shaped built‐in electric field induced by composition variation in the alloy region. The dashed vertical lines mark different electrode positions, allowing a direct comparison of the photocurrent profiles for different channel lengths. For instance, the maxima of the PL gradient and photocurrent are consistent if the electrode is positioned at the center of the built‐in electric field (the origin of the horizontal axis of the main panel).

To evaluate this effect, we collected the current values at two selected positions (indicated by the two dashed lines (pos‐1 and pos‐2) in the main panel of Figure 4) as a function of the laser center position. The resulting line profile of the photocurrent against different electrode positions is plotted in the right panel of Figure 4. At both positions, the maximum photocurrent did not occur when the laser center overlapped with the strongest electric field. In other words, when the electrodes were separated from the center of the built‐in field, the maximum photocurrent was not equivalent to the largest value of the PL peak energy shift, which agreed with the PL and photocurrent mapping measurements (Figure 3c). Instead, the current increased when the laser center began to move toward the electrodes (positive laser center location from the center of the built‐in field). This can be explained by the recombination process of electrons before they reach the electrodes. Thus, the photocurrent peak was not consistently located at the same location as that of the electric field. Furthermore, because pos‐1 was closer to the electric field center than pos‐2, the maximum current value was significantly higher, indicating that a shorter channel length corresponded to a higher photocurrent value. It shall be highlighted that we did not apply a gate voltage to the sample during the measurements, and thus, the electron lifetime of the undoped WSe_2_ or WS_2_ regions outside the alloy region was very short because of the large defect density in typical CVD‐grown samples [32, 33]. In device #1, where the electrodes were far from the alloy region, most of the photogenerated excess electrons recombined before being collected by the electrodes, yielding a photocurrent on the order of pA (Figure S3). In contrast, the shorter channel length of device #2 greatly prevented recombination loss, yielding a photocurrent on the order of nanoamperes (nA) (Figure 3c). The photocurrent peak was closer to the electrodes compared with that to the electric field, which supports the idea of carrier transport and recombination processes (see Figure S4). This behavior also implies a compromise between the excess carrier generation and recombination processes. It is worth noting that only qualitative agreement was obtained from the calculations, since the realistic parameters, such as the electric field distribution, electron lifetime, and mobility, might vary inside the alloy region. Further investigations are necessary to fully exploit the potential of this material for optoelectronic devices.

### 2.4 Performance of Monolayer WS_2x_Se_2(1‐x)_ Photodiode

**FIGURE 5:**
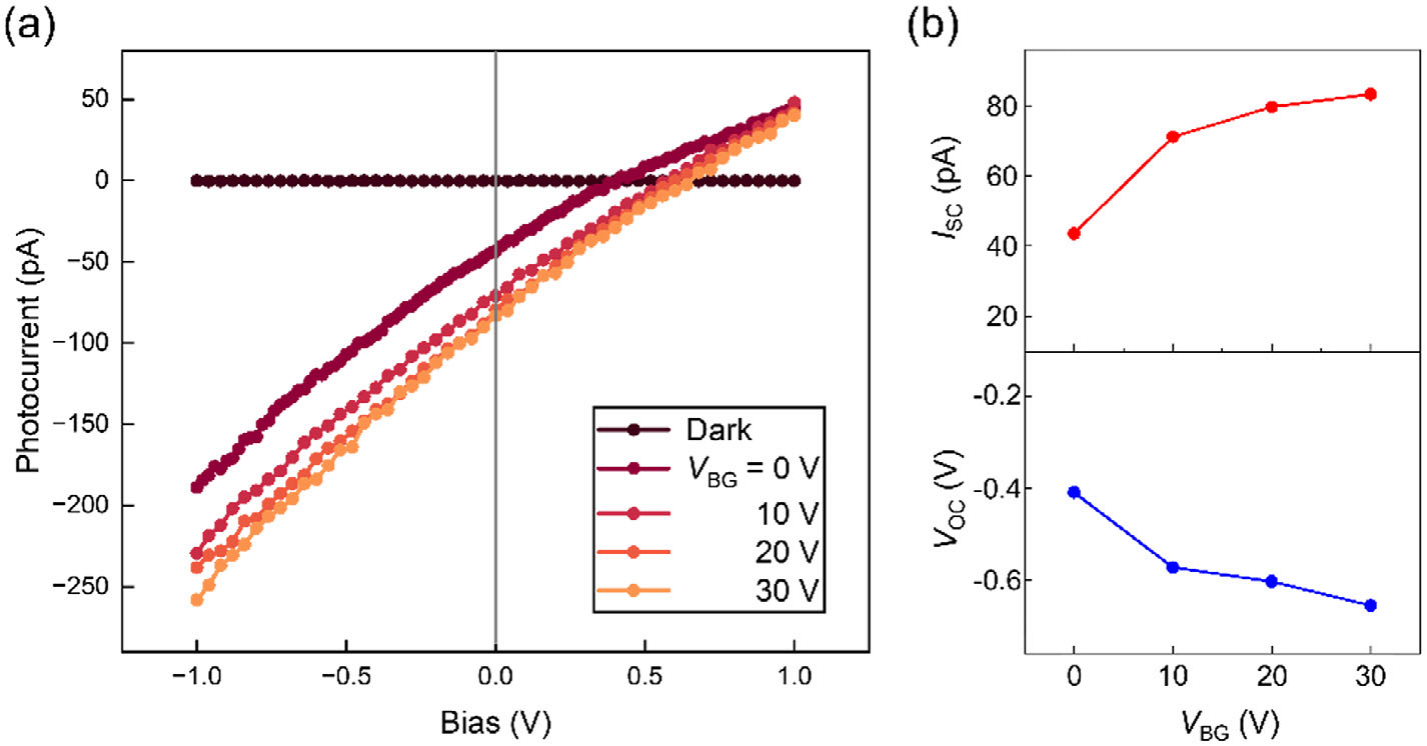
Performance of the monolayer alloy photodiode. (a) Current–voltage (*I*–*V*) curves of the device under different back‐gate voltages. The extracted *I*_SC_ and *V*_OC_ are presented in (b) as a function of the back‐gate voltage *V*_BG_.

Here, we discuss the device performance based on our understanding of the photocurrent generation process. We illuminated the entire channel region of device #2 and measured the *I*–*V* curves. Simultaneously, we applied electron doping through the SiO_2_ back‐gate structure. Under illumination, we obtained a modified *I*–*V* relationship compared with that under dark conditions (Figure 5a). By applying a gate voltage, both *I*_SC_ and *V*_OC_ increased (Figure 5b), similar to those of previously reported photovoltaic devices [34]. Several mechanisms may contribute to this behavior. For example, increased electron doping fills the defect sites, which may prolong the electron lifetime and enhance the mobility, which increases both *I*_SC_ and *V*_OC_. The band bending caused by the gate voltage may also be important in this case.

It is worth noting that the observed *V*_oc_ values of the alloy device (0.41–0.66 V) were generally higher than those reported for pure TMDCs or heterostructure devices without chemical treatments, which were usually below 0.5 V. The comparison is summarized in Section S5. This suggests that a higher energy maximum can be extracted, thereby enabling a higher conversion efficiency [7, 21]. This can be attributed to the significantly wider built‐in field region in the compositionally graded alloy compared with that in lateral heterostructures. At the same photon density, more free carriers can be generated by the field, leading to enhanced device performance. Moreover, owing to the gradually changing composition, the potential barrier height in the alloy region is expected to be significantly lower than that in lateral heterojunctions. In addition, the steep interfaces of lateral heterostructures are more sensitive to defects, which can lead to unexpected band alignment and/or electric field distribution, reducing the device performance. In this regard, the continuous p‐n junction of the alloy may prevent such detrimental effects. For abrupt lateral heterostructures, the depletion/high‐field region is confined to only a few nanometers (nm), and therefore, only a small fraction of photocarriers is generated and efficiently collected at the junction, making the photocurrent highly sensitive to interfacial defects. In compositionally graded alloys, a micrometer‐wide junction can increase the number of photocarriers generated within the junction region. However, for a fixed built‐in potential drop, the reduced average field may reduce the carrier separation/collection efficiency, implying that an optimal junction width is required to attain a balanced carrier generation and field strength. In contrast, the fill factor (FF) of our device was between 22% and 24%, which was lower than the highest reported values for in‐plane heterostructures [18]. This was consistent with the nearly linear *I*–*V* curves (Figure 5a). The relatively low FF might have resulted from the high series resistance, as a moderate gate voltage application significantly increased the *V*_oc_ value. The photovoltaic figures of merit are summarized in Section S6. The external power conversion efficiency (PCE) and external quantum efficiency (EQE) were lower than those reported for state‐of‐the‐art lateral heterostructure photovoltaic devices [17, 18, 19, 35], which was possibly due to carrier collection limitations in the lateral geometry and nonideal transport/contacts. However, a comparatively high VOC indicates a large built‐in potential across the compositionally graded alloy, highlighting its potential for photovoltaic operation upon device optimization. Thus, to further improve device efficiency, it is imperative to optimize the device structure, reduce the contact resistance, and devise synthesis routes that will produce a less defective alloy material. Nevertheless, the 2D nature, spontaneous p‐n junctions, and broad bandgap range make monolayer TMDC alloys promising candidates for simple‐structured photovoltaic applications, including wearable or transparent self‐powered devices and ultrathin solar cells.

## 3 Conclusion

In this study, we demonstrated the photocurrent generation in two‐terminal monolayer WS_2_ *_x_* Se_2(1‐_ *_x_*_)_ alloy devices. KPFM measurements confirmed the presence of a gradual p‐n junction within the compositionally graded alloy region. Simultaneous PL and photocurrent mapping revealed that the observed photocurrent originated from the photovoltaic effect. Furthermore, we demonstrated that the photocurrent generation was unique to the local composition gradient, as supported by the numerical calculations. More importantly, the device based on the monolayer alloy exhibited an open‐circuit voltage as high as 0.66 V. The simple device structure, broadband absorption nature, and high open‐circuit voltage highlight the potential of these devices as efficient optoelectronic devices.

## 4 Methods

### 4.1 PL Mapping Measurements

The PL peak energy mapping measurements were conducted using a laser Raman spectrometer (JASCO NRS‐5100 and HORIBA XploRA PLUS). The laser wavelength and power were 532 nm and 24 µW, respectively. The sample stage was systematically motorized to perform spatial mapping.

### 4.2 KPFM Measurements

KPFM measurements of the surface potential profiles were performed using a Bruker Multimode 8‐HR atomic force microscope. The cantilever employed in the KPFM measurements was SCM‐PIT‐V2 with a Pt/Ir‐coated Si tip. The force constant and resonant frequency were ∼3 N/m and ∼75 kHz, respectively. The data were acquired in amplitude‐modulated KPFM (AM‐KPFM) mode.

### 4.3 Photocurrent Mapping Measurements

To measure the photocurrent generation, photolithography was used to define the electrode pattern, and Au/Ni (50/3 nm) electrodes were deposited. Photocurrent mapping was performed using a Keithley 6514 electrometer. Simultaneously, PL mapping was conducted using a Raman spectrometer with a laser power of 169 µW. For the *I*–*V* measurements, a bias was applied using a Keithley 2612 source meter, and the corresponding photocurrent variation was collected using an electrometer.

## Conflicts of Interest

The authors declare no conflict of interest.

## Supporting information




**Supporting File**: smtd70544‐sup‐0001‐SuppMat.docx

## Data Availability

The data that support the findings of this study are available from the corresponding author upon reasonable request.
