# Supplementary material for: Photovoltaic Device Based on Monolayer Compositionally Graded Transition Metal Dichalcogenide Alloy
Source: Small Methods. 2026 Feb 12;10(5):e01997. doi: 10.1002/smtd.202501997 (PMC12972216; doi:10.1002/smtd.202501997)
Supplement: Supplementary file 1 — Supporting File: smtd70544‐sup‐0001‐SuppMat.docx [file SMTD-10-e01997-s001.docx]

**Supplementary Information**

**Photovoltaic Device Based on Monolayer Compositionally Graded Transition Metal Dichalcogenide Alloy**

Hao Ou,^1*^ Sota Tsukamoto,^2^ Tenta Kitamura,^1^ Motoki Matsuno,^2^ Koshi Oi,^2^ Togo Takahashi,^2^ Takahiro Endo,^3^ Yasumitsu Miyata,^3^ Jiang Pu,^1*^ Taishi Takenobu^2*^

^1^Department of Physics, Institute of Science Tokyo, Tokyo 152-8551, Japan

^2^Department of Applied Physics, Nagoya University, Nagoya 464-8603, Japan

^3^Research Center for Materials Nanoelectronics, National Institute of Materials, Tsukuba 305-0044, Japan

*E-mail: [ou@phys.sci.isct.ac.jp](mailto:ou@phys.sci.isct.ac.jp), [pu@phys.sci.isct.ac.jp](mailto:pu@phys.sci.isct.ac.jp), takenobu@nagoya-u.jp

**S1. Basic characteristics of the compositionally graded monolayer WS_2_*_x_*Se_2(1-_*_x_*_)_ alloy.**


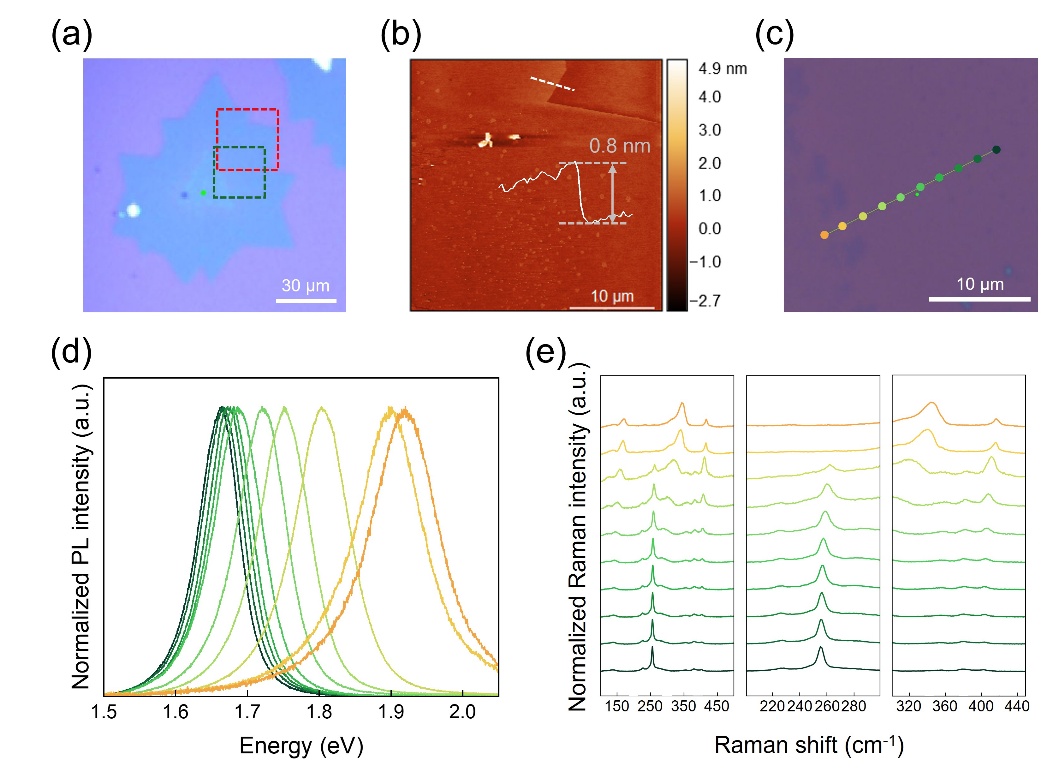


**Figure S1.** Basic characteristics of the compositionally graded monolayer WS_2_*_x_*Se_2(1-_*_x_*_)_ alloy. (a) Microscopic image of the CVD-grown sample. The red and blue rectangles indicate the regions that are subjected to the measurements in the following figures. (b) AFM image of the sample. The linear height variation along the dashed line is shown in the inset. (c) Microscopic image of the region for PL and Raman measurements. The dots along the line indicate the positions of the laser spot in sequential optical measurements. (d) PL and (e) Raman spectra. The colors of the spectra correspond to different positions in (c).

**S2. Fitting of the *I*–*V* curve using the modified Shockley-like diode equation**

In a *p*-*n* junction, where the composition changes continuously, the built-in field deviates from the conventional constant, as defined in the textbook. However, as derived in [1], the current–bias relationship still obeys the Shockley-like diode equation, which is expressed as $\begin{aligned} I=I_{s}^{'}\left( \exp\left( \frac{qV}{kT} \right)-1 \right),\#\left( S1 \right) \end{aligned}$

where *V* is the bias and *q*, *k*, and *T* represent the elementary charge, Boltzmann constant, and temperature, respectively. $I_{s}^{'}$ is the modified saturation current, which is given by $\begin{aligned} I_{s}^{'}=\frac{qD_{n}n_{in}^{2}}{\int_{0}^{W_{n}} p_{n}\left( x \right)dx}+\frac{qD_{p}p_{ip}^{2}}{\int_{0}^{W_{p}} n_{p}\left( x \right)dx}.\#\left( S2 \right) \end{aligned}$

Here, *D*_n_ and *D*_p_ denote the electron and hole diffusion coefficients, respectively. *n*_in_ and *p*_ip_ represent the intrinsic electron and hole concentrations in the *n*-type and *p*-type regions, respectively. *p*_n_ and *n*_p_ are the minor carrier concentrations in the *n*-type and *p*-type regions, respectively. *W*_n_ and *W*_p_ are the lengths of the compositionally graded regions.

To include the influence of the series resistance, we further modified Equation (1) as^[2]^ $\begin{aligned} I=\frac{nkT}{qR_{s}}W\left[ \frac{I_{s}^{'}R_{s}q}{nkT}\mathrm{ex}p \left( \frac{V+I_{s}^{'}R_{s}}{n}\cdot\frac{kT}{q} \right) \right]-I_{s}^{'}.\#\left( S3 \right) \end{aligned}$

Here, *R*_s_ is the series resistance, *n* represents the ideality factor, and *W* is the Lambert W function. We fitted the *I*–*V* curve of device #1 under illumination, as shown below:


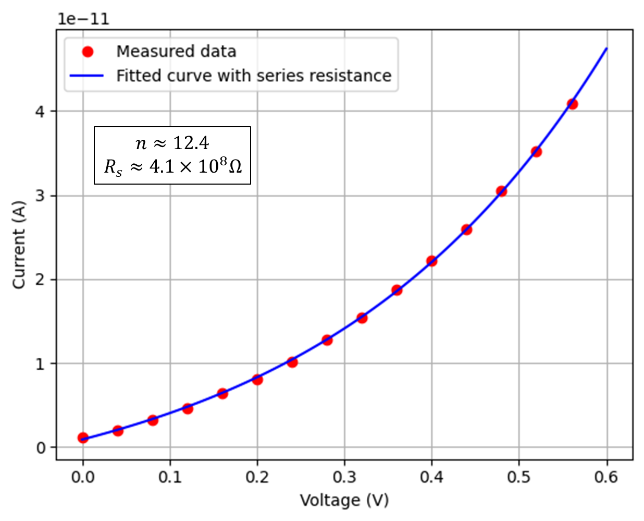


**Figure S2**. Fitting of the *I*–*V* curve for device #1 under illumination.

**S3. Results of the photoluminescence and photocurrent mapping for device #1**


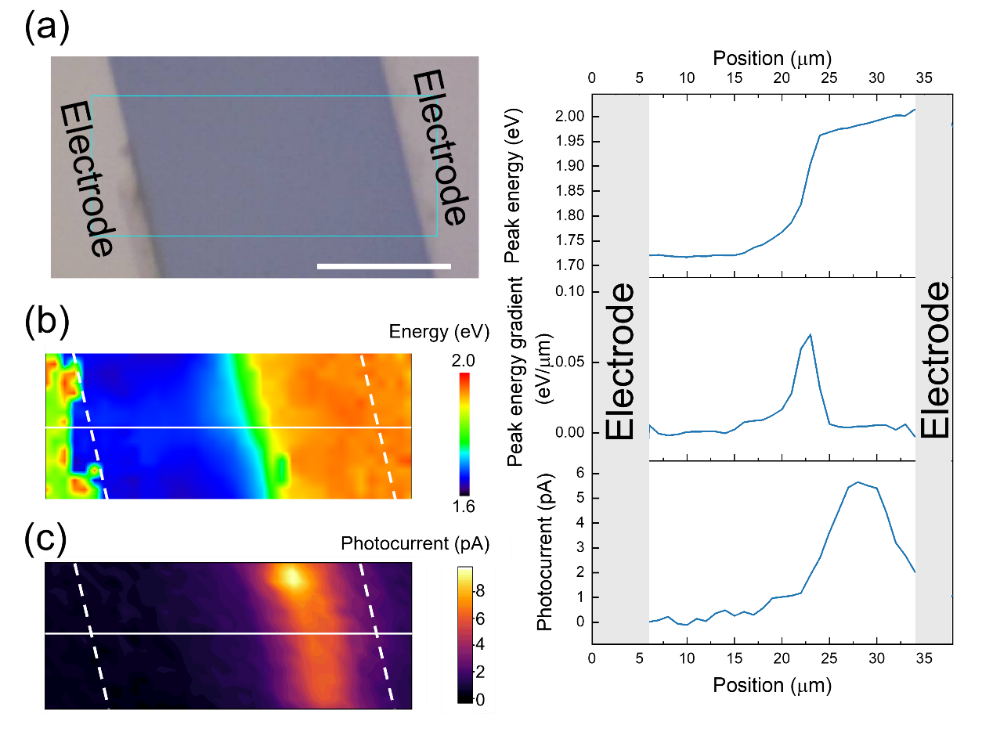


**Figure S3.** Photocurrent generation characterization of the monolayer alloy device. (a) Optical image of device #1. The scale bar is 10 μm. (b) PL map of the channel region. (c) Corresponding photocurrent map. (d) Peak energy, peak energy gradient, and photocurrent profiles along the diagonal dashed white lines in (b) and (c).

**S4 Details of the photocurrent calculation**

　We numerically studied the photocurrent generation behavior of the alloy by considering the continuity equation of the carrier (electron) density *n*, which is expressed as $\begin{aligned} \frac{dn}{dt}=G+\frac{1}{q}\frac{d{(J}_{drift}+J_{diffusion})}{dx}-\frac{n}{\tau}.\#\left( S4 \right) \end{aligned}$

Here, *G* is the generation rate of electrons, and *J*_drift_ and *J*_diffusion_ are the drift and diffusion currents, respectively. *τ* is the lifetime. Specifically, for the current terms, we have $\begin{aligned} J_{drift}=\mu n\frac{dE}{dx}+\mu\frac{\partial n}{\partial x}E,\mathrm{and}\#\left( S5 \right) \end{aligned}$ $\begin{aligned} J_{diffusion}=D\frac{\partial n}{\partial x},\#\left( S6 \right) \end{aligned}$

where *μ* is the electron mobility, *E* is the built-in field, and *D* is the electron diffusion coefficient. To calculate the photocurrent at equilibrium, we set d*n*/d*t* to zero and solved for *n*. Finally, the generated current *J* = *J*_drift_ + *J*_diffusion_ was calculated. For the parameters, we set *τ* = 20 ps based on a previous study.^[3]^ We assumed that the electron had a diffusion length of *L*_D_ = ~100 nm. Hence, we could calculate *μ* and *D* using the relationships

$\begin{aligned} \mu=\frac{{L_{D}}^{2}}{\tau},\#\left( S7 \right) \end{aligned}$ $\begin{aligned} D=\frac{{L_{D}}^{2}}{kT\tau}.\#\left( S8 \right) \end{aligned}$

Here, *k* is the Boltzmann constant and *T* is 300 K. Specifically, we let *G* possess a Gaussian form, with a standard deviation of *σ*_G_ = 2 μm to mimic the laser illumination. We also set a Gaussian geometry for the electric field *E*, with a maximum value of *E*_max_ = 10^4^ V/m and standard deviation of *σ*_E_ = 3 μm. To test the photocurrent mapping, we shifted the laser center (i.e., the location of *G*) from the left side of *E* to the right side. We performed the calculations in a one-dimensional manner.

The photocurrent map is shown in Figure 4 of the main text. Here, we present the photocurrent map again and compare the photocurrent values when the laser center is at different locations. The results are shown in Figure S4. Apparently, when the laser center was at a location with a higher electric field strength, a higher maximum current was generated. However, when the laser center was closer to the position slightly away from the center (gray dashed line in Figure S4b), a higher photocurrent was generated compared with that when the laser center was at the maximum electric field strength but farther from the position. This explains why the peak of the photocurrent is closer to the electrode than the peak of the PL peak energy gradient, which we regard as an index of the local electric field strength.


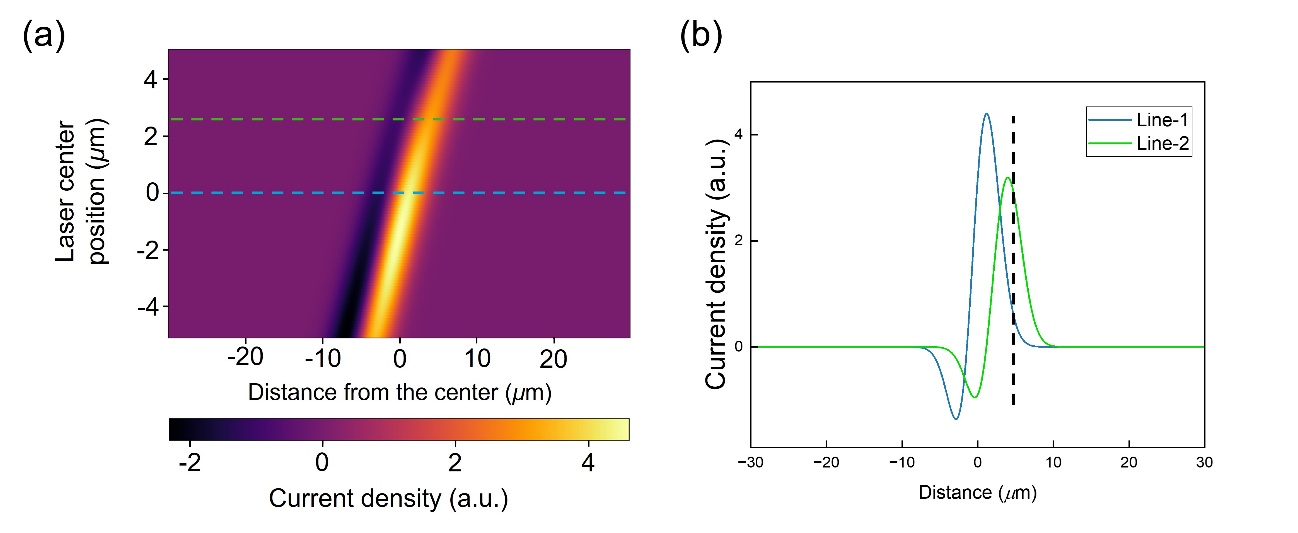


**Figure S4.** Calculation of the photocurrent density. (a) Calculated distance and laser position dependence of the photocurrent density. The current density profiles along the two horizontal dashed lines are shown in (b). Even though the maximum value is higher when the laser position is at the center of the electric field, its value will decrease rapidly when the distance increases, and will be inferior to the value when that laser position is closer to the given distance (vertical dashed line in (b)).

**S5. Comparison of the open-circuit voltage values**

| Device material | *p*-*n* junction formation type | Illumination condition | Highest *V*_OC_ (V) | Ref. |
| --- | --- | --- | --- | --- |
| Monolayer WS_2_*_x_*Se_2(1-_*_x_*_)_ | Gradual lateral heterojunction | 532-nm laser | 0.44–0.66 | This work |
| Few-layer WSe_2_ | Homojunction (O_2_ plasma-treated) | 532-nm laser | 0.26 | [4] |
| Few-layer WS_2_ | Graphene/WS_2_/graphene heterojunction | 488-nm laser | 0.14 | [5] |
| Monolayer WSe_2_ | Split-gated homojunction | White light | 0.80 | [6] |
| Monolayer WSe_2_ | WSe_2_/h-BN/graphene heterojunction | 638-nm laser | 0.83 | [7] |
| 1L WSe_2_/3L WSe_2_ | Layer engineering | 632-nm monochromatic light | 0.11 | [8] |
| Lateral WS_2_/WSe_2_ | Lateral heterojunction | 514-nm laser | 0.47 | [9] |
| Lateral WS_2_/MoS_2_ | Lateral heterojunction | 532-nm laser | 0.32 | [10] |
| Multilayer WSe_2_/InSe | van der Waals heterojunction | 532-nm laser | 0.22 | [11] |
| Few-layer WSe_2_/ MoS_2_ | van der Waals heterojunction | 633-nm laser | 0.35 | [12] |
| Few-layer n-MoS_2_/  p-MoS_2_ | Chemical doping | 660-nm monochromatic light | 0.45 | [13] |
| Few-layer n-MoS_2_/  p-MoS_2_ | Chemical doping | Halogen | 1.02 | [14] |

**S6. Summary of the photovoltaic parameters of the device**

| Short-circuit current density, *J*_SC_ (mA/cm^2^) | Fill factor, FF (%) | Power conversion efficiency, PCE (%) | Equivalent quantum efficiency, EQE (%) |
| --- | --- | --- | --- |
| 64  (focused beam)  0.014–0.026  (entire channel) | 22–24  (entire channel) | 1.8–5.1 (×10^-5^)  (entire channel) | 0.02  (focused beam)  4.2–8.1 (×10^-4^)  (entire channel) |

**References (SI)**

[1] L. Zhang, H. Wu, C. He, K. Zhang, Y. Liu, Q. Wang, L. He, W. Zhao, Z. Chen, *Micromachines* **2024**, *15*, 778.

[2] J.-W. Chen, S.-T. Lo, S.-C. Ho, S.-S. Wong, T.-H.-Y. Vu, X.-Q. Zhang, Y.-D. Liu, Y.-Y. Chiou, Y.-X. Chen, J.-C. Yang, Y.-C. Chen, Y.-H. Chu, Y.-H. Lee, C.-J. Chung, T.-M. Chen, C.-H. Chen, C.-L. Wu, *Nat. Commun.* **2018**, *9*, 3143.

[3] M. Shimasaki, T. Nishihara, N. Wada, Z. Liu, K. Matsuda, Y. Miyata, Y. Miyauchi, *Appl. Phys. Express* **2023**, *16*, 012010.

[4] S. B. Mitta, F. Ali, Z. Yang, I. Moon, F. Ahmed, T. J. Yoo, B. H. Lee, W. J. Yoo, *ACS Appl. Mater. Interfaces* **2020**, *12*, 23261.

[5] L. Britnell, R. M. Ribeiro, A. Eckmann, R. Jalil, B. D. Belle, A. Mishchenko, Y.-J. Kim, R. V. Gorbachev, T. Georgiou, S. V. Morozov, A. N. Grigorenko, A. K. Geim, C. Casiraghi, A. H. C. Neto, K. S. Novoselov, *Science* **2013**, *340*, 1311.

[6] A. Pospischil, M. M. Furchi, T. Mueller, *Nat. Nanotechnol.* **2014**, *9*, 257.

[7] D. Li, M. Chen, Z. Sun, P. Yu, Z. Liu, P. M. Ajayan, Z. Zhang, *Nat. Nanotechnol.* **2017**, *12*, 901.

[8] Z. Wang, Y. Chen, P. Wu, J. Ye, M. Peng, Y. Yan, F. Zhong, T. He, Y. Wang, M. Xu, K. Zhang, Z. Hu, Q. Li, L. Zhang, F. Wang, P. Wang, *Infrared Phys. Technol.* **2020**, *106*, 103272.

[9] X. Duan, C. Wang, J. C. Shaw, R. Cheng, Y. Chen, H. Li, X. Wu, Y. Tang, Q. Zhang, A. Pan, J. Jiang, R. Yu, Y. Huang, X. Duan, *Nat. Nanotechnol.* **2014**, *9*, 1024.

[10] W. Wu, Q. Zhang, X. Zhou, L. Li, J. Su, F. Wang, T. Zhai, *Nano Energy* **2018**, *51*, 45.

[11] B. Xu, Y. Li, Z.-Y. Sun, Z. Zhao, L. Yang, F. Gao, P.-A. Hu, L. Zhen, C.-Y. Xu, *Adv. Electron. Mater.* **2021**, *7*, 2100584.

[12] J. Wong, D. Jariwala, G. Tagliabue, K. Tat, A. R. Davoyan, M. C. Sherrott, H. A. Atwater, *ACS Nano* **2017**, *11*, 7230.

[13] S. A. Svatek, E. Antolin, D.-Y. Lin, R. Frisenda, C. Reuter, A. J. Molina-Mendoza, M. Muñoz, N. Agraït, T.-S. Ko, D. P. de Lara, A. Castellanos-Gomez, *J. Mater. Chem. C* **2017**, *5*, 854.

[14] S. A. Svatek, C. Bueno-Blanco, D.-Y. Lin, J. Kerfoot, C. Macías, M. H. Zehender, I. Tobías, P. García-Linares, T. Taniguchi, K. Watanabe, P. Beton, E. Antolín, *Nano Energy* **2021**, *79*, 105427.
